# Comparison of murine retroorbital plexus and facial vein blood collection to mitigate animal ethics issues

**DOI:** 10.1186/s42826-021-00090-4

**Published:** 2021-05-06

**Authors:** Eun Jung Jo, Eunjin Bae, Jeong-Hwan Yoon, Ji Yeon Kim, Jin Soo Han

**Affiliations:** 1 Department of Laboratory Animal Medicine, College of Veterinary Medicine, Konkuk University, Seoul, 05029 Republic of Korea; 2 Biomedical Research Institute, Kyungpook National University Hospital, Daegu, 41944 Republic of Korea

**Keywords:** Serial blood collection method, Facial vein, Retroorbital plexus, Animal ethics

## Abstract

**Background:**

Blood collection is an important procedure used in animal experiments. Blood collection methods that reduce pain, injury, and stress in experimental animals are important with regard to animal ethics. Various comparative studies of blood collection methods have been reported; however, there are no comparative studies on serial blood collection considering animal ethics. To suggest simple methods that minimize pain during serial blood collection, we compared the retroorbital plexus (RP) and facial vein (FV) blood collection methods performed by both experienced and novice groups. The experienced and novice groups collected up to 0.4 mL of blood via the RP and FV methods every second day for 2 weeks. After blood collection, all mice were evaluated by corticosterone concentrations for stress, hematological, immunological, and histological analyses.

**Results:**

We found that the FV methods reduced the collection time, pain, distress, tissue damage and lasting harms without anesthesia. Corticosterone concentrations in the peripheral blood were decreased in mice subjected FV methods compare with those subjected to RP methods. The proportion of granulocytes and monocytes, such as macrophages in the peripheral blood and spleen, was decreased in mice subjected to FV methods compared with that in mice subjected to RP methods in both experienced and novice groups. White blood cells were infiltrated in RP areas with severe tissue damage and inflammation.

**Conclusions:**

With respect to animal ethics, we suggest that the FV method, a simple and fast technique that can easily be performed by both experienced and novice researchers, is suitable for serial blood collection.

## Background

Blood collection is one of the basic methods used to obtain the experimental results from in vivo studies. The quantity and quality of blood extracted varies according to the experimental aim and study design, which can affect experimental results [1]. When researchers plan to undertake blood collection, they should consider not only the methods but also animal welfare, with the aim of minimizing pain, distress, tissue damage, and lasting harm to the animal [2, 3].

There are multiple sites for blood collection in mice, including the retroorbital plexus (RP), lateral tail vein, saphenous vein, heart, and facial vein (FV), also known as the submandibular vein [3]. The RP method is widely performed using capillary tubes to draw blood from the retrobulbar venous sinus. It can easily yield a large amount of blood in a short period of time; however, multiple side effects including hematomas, damage to surrounding tissues, periosteum, gland hardening, orbital bone fracture, and inflammation of eye muscles in the blood collection area have been observed [4–7]. RP blood collection in rats can lead to abnormalities, depending on the skill level of the technician [1, 5]. Recently, most Institutional Animal Care and Use Committees (IACUCs) and the National Institutes of Health have disallowed RP blood collection without anesthesia because of the severe risks to the eyes of mice [3, 8, 9].

The FV method involves puncturing the FV located in the lower jaw using a lancet. It is possible to acquire up to 0.7 mL blood through this method; after collection, the bleeding can be stopped by applying light pressure to the area from which blood was drawn [10–13]. This is a rapid and simple method for collecting blood from unanesthetized mice. It reduces stress, pain, and damage to the inner ear, facial muscles, and nerves of mice. FV blood collection also improves the animal welfare practices in experimental protocols [14]. The FV method is a suitable alternative to the RP; however, the advantages and disadvantages of each methods remain controversial [15]. Both FV and RP blood collection methods can cause severe tissue damage and trauma. Blood can be collected from multiple muscles, nerves, and blood vessels; the skills developed through experience are essential for animal welfare.

Although several studies have compared the FV and RP methods, most studies utilized only one or two blood samples and did not address the potential for tissue damage caused by serial RP blood collection [12, 16–19]. Comparative analyses of the FV and RP methods have reported the effects on stress and tissue damage, but to our knowledge, no comparative study that assesses the effects of the skill levels of experienced and novice researchers has been conducted [15].

In this study, the FV method was found to be suitable for minimizing the adverse clinical effects of tissue damage and inflammation without anesthesia. Furthermore, despite serial blood collections, the FV method caused significantly reduced tissue damage, corticosterone concentrations in the peripheral blood, and expression of immune cells in the peripheral blood and spleen compared with those by the RP method, for both experienced and novice researchers. We suggest that FV blood collection should be the preferred method for short-term and mid-term mouse experiments requiring serial collections with regard to ethical considerations.

## Results

### FV blood collection is faster than RP blood collection

**Fig. 1:**
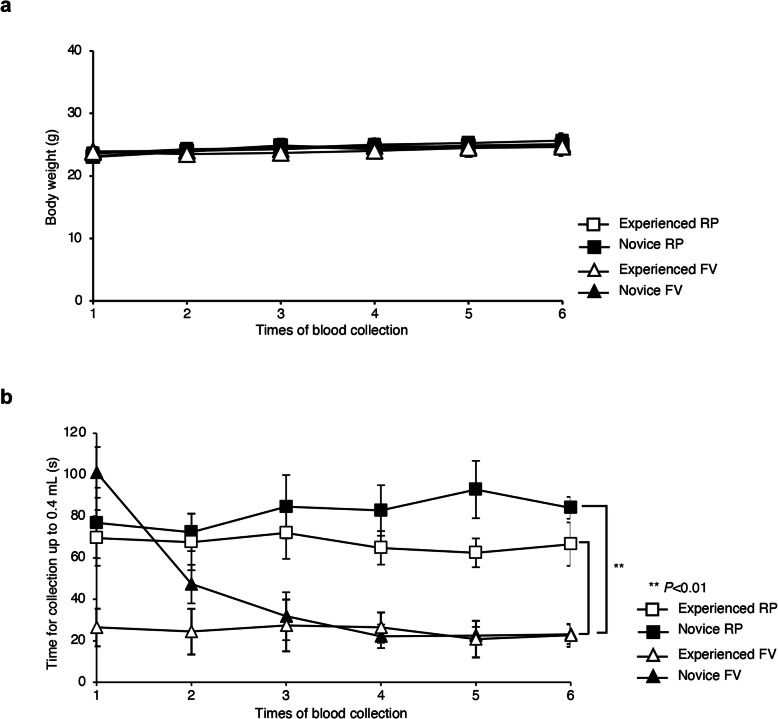
Body weights and blood collection times. **a** Body weights of blood collection method in all groups (*n* = 6/blood collection method) were measured before blood collection. **b** Blood collection times for the retroorbital plexus (RP) and facial vein (FV) methods for experienced and novice groups. Data are shown as means ± s.d. *P* values were calculated by two-way ANOVA between the RP and FV methods, with blood collection method in all groups (n = 6/blood collection method). **P < 0.01

To assess simplicity and proficiency, we divided RP and FV blood collection into two groups of “experienced” and “novice” researchers, based on their level of experience with animal experimentation. Before commencing blood collection, we measured the body weight of the mice. Their body weights increased slightly over the course of serial collections; however, no significant changes were observed between the RP and FV blood collection mice in all groups at any time (Fig. 1a).

We next recorded the time taken to collect 0.4 mL blood by both groups. In the FV blood collection performed by both experienced and novice researchers, except for a first-time novice, was faster than RP blood collection. The acquisition of skills for FV blood collection by the novice group improved their speed after the second collection, whereas the time required for the FV blood collection decreased for the experienced group at the third collection (Fig. 1b). In contrast, the time required for RP blood collection was significantly increased in novice groups than experienced researchers throughout the experimental period (Fig. 1b). These results show that the blood collection time of the FV method was shorter than that of the RP method for both experienced and novice groups.

### RP blood collection induces higher corticosterone levels

**Fig. 2:**
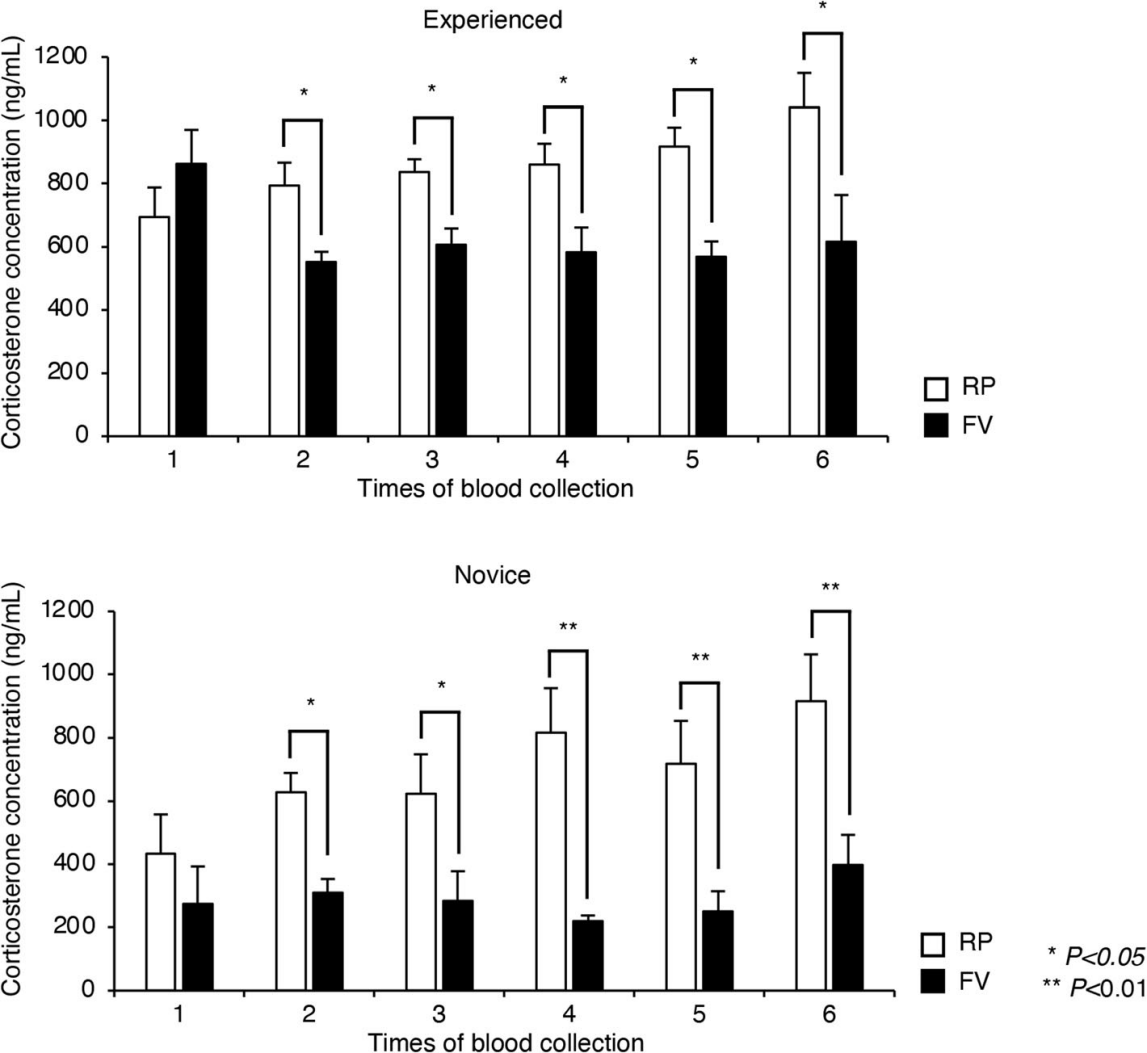
Corticosterone concentrations in the peripheral blood. Corticosterone concentrations in the peripheral blood of mice subjected to the retroorbital plexus (RP) and facial vein (FV) methods were measured by ELISA. Data are shown as means ± s.d. *P* values were calculated by two-tailed unpaired Student’s *t*-test, with n = 6/blood collection method. *P < 0.05 and **P < 0.01

We measured the corticosterone concentration of blood serum to evaluate the distress of mice during the experimental periods though ELISA. Corticosterone concentration of mice subjected to the RP method increased for both the experienced and novice groups over the experimental period. Serum corticosterone levels were significantly lower for the FV method than for the RP method for both researcher groups (Fig. 2). This result indicates that the stress levels of the mice subjected to the FV method were lower than those of the mice subjected to the RP method.

### Changes in immune cell proportions in the peripheral blood and spleen

The corticosterone concentration in the peripheral blood was significantly decreased in mice subjected to the FV method in both experienced and novice groups. To determine the correlation between corticosterone and blood cells, we next compared cell-count changes in the peripheral blood between mice subjected to the RP and FV methods using an auto hematology analyzer. The proportions of white blood cells (WBCs) in the blood of mice subjected to the RP method were significantly increased at the sixth collection. Especially, the proportions of granulocytes and monocytes were also increased at sixth collection. No differences were observed with the FV method (Table 1).

**Table 1:** Changes in cell counts in the peripheral blood

a. RP		
**Times of blood collection**	**1**	**6**
WBC	4.8(±0.2)×10^3^/μL	7.8(±1.2)×10^3^/μL**
Lymphocytes	3.7(±0.3)×10^3^/μL	5.0(±0.1)×10^3^/μL
Monocytes	0.1×10^3^/μL	0.3(±0.05)×10^3^/μL*
Granulocytes	1.0(±0.2)×10^3^/μL	2.5(±0.2)×10^3^/μL**
Lymphocytes (%)	75.80(±1.2)%	69.20(±3.2)%
Monocytes (%)	3.10(±0.3)%	4.20(±0.8)%*
Granulocytes (%)	21.10(±1.0)%	32.60(±2.5)%**
RBC	7.70(±0.54)×10^6^/μL	7.26(±0.53)×10^6^/μL
HGB	13.8(±0.5)g/Dl	13.1(±0.2)g/Dl
**b. FV**		
**Times of blood collection**	**1**	**6**
WBC	5.9(±1.8)×10^3^/μL	4.7(±2.3)×10^3^/μL
Lymphocytes	4.1(±2.3)×10^3^/μL	3.1(±1.2)×10^3^/μL
Monocytes	0.2(±0.05)×10^3^/μL	0.2(±0.1)×10^3^/μL
Granulocytes	1.6(±0.4)×10^3^/μL	1.4(±0.3)×10^3^/μL
Lymphocytes (%)	69.70(±3.2)%	67.00(±1.2)%
Monocytes (%)	3.30(±0.3)%	3.90(±0.5)%
Granulocytes (%)	27.00(±1.0)%	29.10(±0.3)%
RBC	9.13(±0.13)×10^6^/μL	9.68(±0.43)×10^6^/μL
HGB	12.6(±0.4)g/Dl	13.2(±1.2)g/Dl

Peripheral blood samples from the RP (a) and FV (b) methods were measured using a fully automatic hematology analyzer. Data are shown as means ± s.d. *P* values were calculated by the two-tailed unpaired Student’s *t*-test, with blood collection method in all groups (*n* = 6/blood collection method). *P < 0.05 and **P < 0.01

**Fig. 3:**
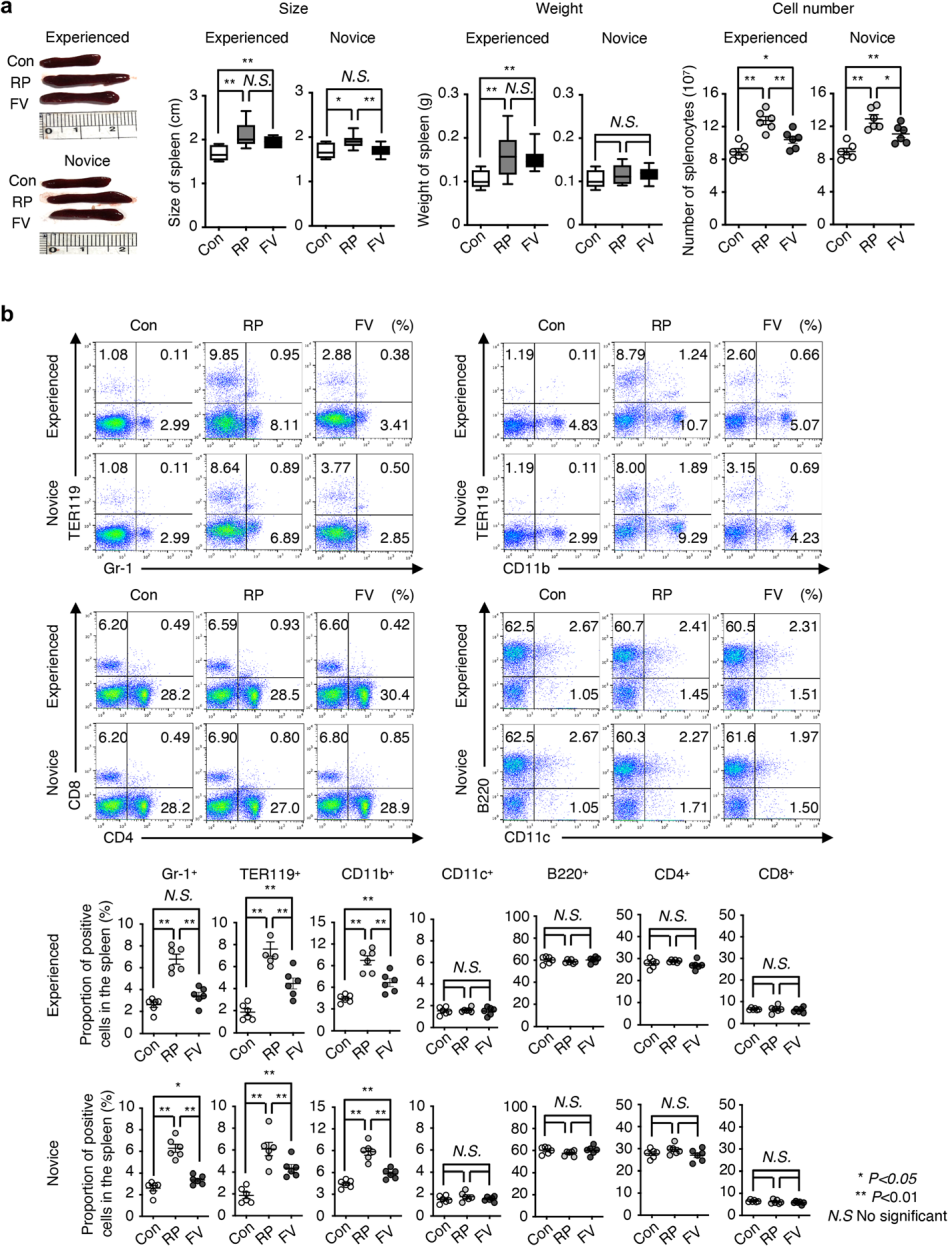
Changes in immune cell levels in the spleen. **a** Difference in sizes, weights, and cell numbers in mouse spleens as a function of blood collection method and researcher experience. **b** Immunophenotyping of RP and FV blood collection mice by flow cytometry. Dot plots show the expression of TER119, Gr-1, CD11b, CD11c, B220, CD8, and CD4 in the spleen. Graphs show proportions of cell populations in the spleen. Data are shown as means ± s.d. *P* values were calculated by two-tailed unpaired Student’s *t*-test, with blood collection method in all groups (n = 6/blood collection method). *P < 0.05, **P < 0.01, and *N.S* not significant

We then examined the differences in immune cells in the spleen of mice subjected to the RP and FV methods. Size, weight, and cell numbers were significantly increased in mice subjected to the RP method for both experienced and novice groups compared with those in the control mice. Interestingly, the size and cell numbers were significantly decreased in the mice subjected to the FV method for the novice group compared with those in mice subjected to the RP method (Fig. 3a). We confirmed that the proportions of immune cells in the spleen (granulocytes as Gr-1^+^, erythrocytes as TER119^+^, and macrophages as CD11b^+^) were significantly increased in mice subjected to both methods for both researcher groups compared with the control mice. The proportions of Gr-1^+^, TER119^+^, and CD11b^+^ cells were significantly decreased in mice subjected to the FV method in both experienced and novice groups compare to those of mice subjected to the RP method (Fig. 3b). There were no significant differences in the proportions of CD11c^+^, B220^+^, CD4^+^, and CD8^+^ cells between the experienced and novice groups throughout the study. These results showed that the proportion of granulocytes and macrophages in both peripheral blood and spleen was higher in mice subjected to the RP method than in those subjected to the FV method.

### Tissue damage associated with RP blood collection

**Fig. 4:**
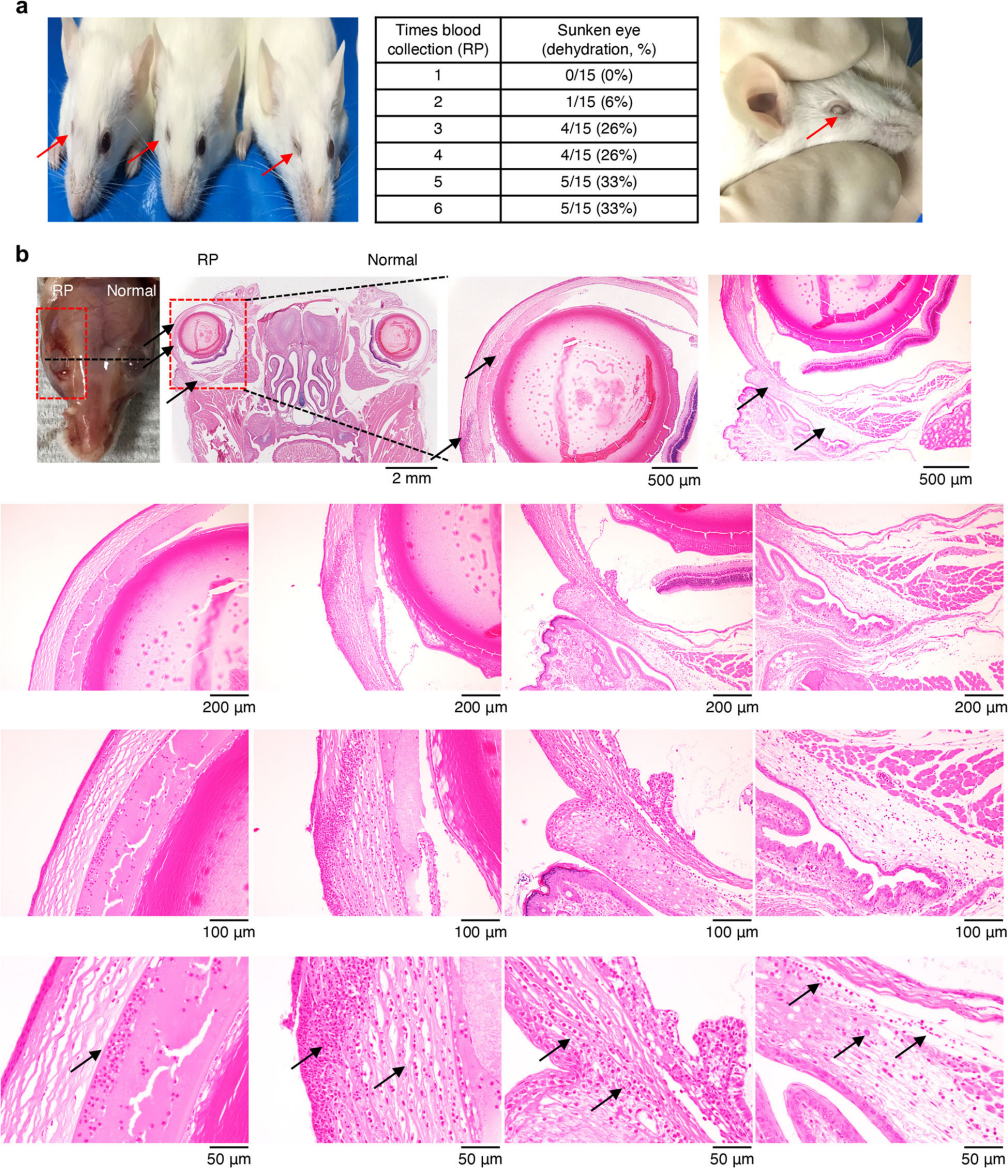
Clinical and histological effects of retroorbital plexus (RP) blood collection. **a** Proportions of sunken eyes (left) and cataracts (right). **b** Histology of ocular tissues (hematoxylin and eosin, magnification × 10, × 40, × 100, × 200, and × 400, scale bar: 2 mm, 500 μm, 200 μm, 100 μm, and 50 μm). Arrows indicate infiltrations of red blood cells, mononuclear cells, monocytes, and granulocytes such as neutrophils

WBC counts, especially those of granulocytes and macrophages, were significantly increased in the blood and spleens of mice subjected to the RP method. We then performed histological analysis of the RP regions to assess the clinical damage. When the RP method was performed by novice groups, four mice showed sunken eyes at the third collection. At the fifth collection, severe dehydration was observed in five mice (Fig. 4a, left and middle). Furthermore, the eyes of the mice had not recovered at 5 days after blood collection. Cataracts were observed in all mice damaged by the RP method (Fig. 4a, right), and hemorrhage in the orbital region of the right eye was identified compared with the left eye (Fig. 4b). Hematoxylin and eosin staining of ocular tissue showed that red blood cells, mononuclear cells, monocytes, and granulocytes including neutrophils, infiltrated the connective tissues of the cornea and RP blood collection sites (Fig. 4b).

## Discussion

Blood collection is essential for generating data in laboratory animal experiments. It is important to obtain the desired amount of blood using simple techniques with a short collection time while causing minimal pain and stress to the animals [2, 20, 21]. Recently, several studies have investigated animal ethics and welfare practices for experimental animals by comparing multiple blood collection methods [3, 15].

RP blood collection is typically performed at contract research organizations, pharmaceutical companies and academic institutions [22]; however, severe side effects, including hematomas, damage to surrounding tissues, periosteum, gland hardening, orbital bone fracture, and inflammation of muscle in the blood collection area have been observed [20, 23]. In contrast, FV blood collection has only recently become common. This method reduces stress, pain, and damage to the inner ear, facial muscles, and nerves of mice. FV blood collection improves animal welfare practices in experimental protocols [14].

Both the RP and FV methods have been widely used in animal experiments, but adverse clinical effects and lasting harm to mice remain important issues. The RP method is used frequently, but as per the guidelines of most IACUCs, anesthesia is required while performing RP blood collection. Compared to other blood collection methods, RP blood collection has a variety of variables that must be considered [5]. In contrast, the FV method can be used without anesthesia, which is a major advantage among various blood collection methods.

This study was conducted to propose methods that minimize adverse effects in mice and promote animal welfare practices. In a previous study, the procedures were conducted by experienced researchers only [19]. However, to better evaluate the methods and determine how quickly skills could be acquired to perform RP and FV methods, we divided the individuals performing the collections into experienced and novice researcher groups.

The RP method with anesthesia required more time to complete than the FV method for both experienced and novice researchers. For novices, the first collection time was longer for FV method than for the RP method; however, with successive collections, the FV collection time of novice researchers were significantly decreased during repeating time (Fig. 1b). These results suggest that novice researchers experienced difficulty with animal handling techniques and in identifying correct anatomical positions for blood collection without anesthesia during the first collection. Novice researchers acquired more skill with the FV method over repeated collections. Thus, the time of blood collection decreased steadily.

Corticosterone concentrations in the peripheral blood were increased in mice subjected to the RP method compare with those subjected to the FV method for both experienced and novice researchers, correlating with the time taken to collect the blood. This indicates that the pain and stress of mice subjected to the RP method increased with collection time. It is well known that anesthesia and other environmental conditions, such as noise and temperature, can also cause stress in animals [3, 24, 25].

Differences in pain and stress were indicated by changes in the WBC counts in the peripheral blood and immune organs. Increased in granulocytes, monocytes, and lymphocytes are correlated with anxiety, stress, and oxidative damage [26, 27]. The proportion of granulocytes and monocytes in the peripheral blood and the proportion of Gr-1^+^, Ter119^+^, and CD11b^+^ cells in the spleen were increased in the mice subjected to RP method. Higher pain and stress levels in mice subjected to the RP method affect immune cell expression in the blood and immune organs.

Although experienced researchers can routinely obtain large quantities of blood by the RP method, the pain and stress cause to the animals are considerable, even with anesthesia. Therefore, it is necessary to consider how blood collection can be made less stressful for the animal while satisfying both researcher requirements and animal ethical guidelines.

According to the guidelines for the health evaluation of experimental laboratory mice, damaged and sunken eyes are classified as Level 4: severe pain and distress [2]. We found that mice serially subjected to RP method showed ocular lesions, enophthalmos, cataracts and dehydration with hemorrhages in the orbital region. Severe inflammation, particularly the infiltration of neutrophils, monocytes and lymphocytes, was observed in the connective tissues of the cornea and puncture regions. The damaged eyes did not recover during serial RP blood collections. We conclude that it is better to avoid the RP method or change to the FV method for serial blood collection, as required in studies involving diabetes, obesity, and other metabolic diseases.

## Conclusions

FV blood collection reduces collection time, tissue damage, corticosterone level in the peripheral blood and decreases immune cell populations in the peripheral blood and spleen relative to those with the RP method for both experienced and novice researchers. Our findings suggest that the FV method is more suitable for serial blood collection and mitigates animal ethics concerns because it is a simple and fast technique.

## Methods

### Experimental animals

Age-matched female ICR mice (6-week-old, *n* = 72) were purchased from Koatech Inc. (Pyeongtaek, Gyeonggi, Korea). Mice were maintained at the Experimental Animal Center in College of Veterinary Medicine (Konkuk University, Seoul, Korea) under specific pathogen-free (SPF) conditions and a constant 12 h strict dark-light cycle, 22 ± 2 °C room temperature, and 50 ± 10% relative humidity. After a week of acclimatization, mice were divided into two blood collection methods (RP and FV, *n* = 6/collection method) in each researcher. The experimental protocol was approved according to the ethical guidelines of the Institutional Animal Care and Use Committee of Konkuk University, approval No. KU18190.

### Researchers

The guidelines were reviewed with both experienced and novice researchers, with the data from each researcher group kept separate. Experienced researchers had experience in animal experimentations and had conducted both RP and FV blood collection previously. Novice researchers were graduate students who took classes on laboratory animals but had no prior experience in animal experimentations. RP and FV blood collection techniques were performed by three researchers per group.

### Anesthesia

Isoflurane liquid (Ifran, Hana Pharm Co., Ltd., Korea) was applied to the anesthesia chamber with oxygen placed in a certified ducted hood. This method was used only for RP blood collection, and blood was collected after confirming muscle relaxation and stable breathing.

### Blood collection

Mice were weighed before all collections. RP blood collection was performed under anesthesia by inserting a micro-hematocrit capillary tube (HCH-42A2502, KIMBLE CHASE, USA) into the venous sinus behind the eyeball and exerting pressure to penetrate the sinus. FV blood collection was performed by stabbing an animal disposable blood lancet (GR 4MM, MEDIpoint Inc., USA) into the top rear side of the submandibular jawbone, where veins are gathered. Blood was collected in a collection tube (365992, BD Microtainer BD bioscience, USA). The collection time was recorded from anesthesia time (30 s) to when 0.4 mL had been extracted.

### ELISA for corticosterone

An ELISA Kit (ADI-900-097, Enzo Life Sciences Inc., USA) was used to measure corticosterone concentrations according to the manufacturer’s instructions. All samples were analyzed in triplicate. Alkaline phosphatase conjugated with corticosterone was inserted into each sample. Next, mouse monoclonal antibody was attached to corticosterone and the sample was incubated at room temperature for 2 h. Samples were washed three times, and p-nitrophenyl phosphate was added in each well. After 1 h, trisodium phosphate was added, and the plate was read at an optimal absorbance of 405 nm using a microplate reader (SpectraMAX 190, Molecular Devices, USA).

### Hematological analysis

For each group, 0.1 mL whole blood was stored separately in a blood collection tube (BD Bioscience). Tubes were inverted to mix the blood immediately to prevent clots and platelet clumps. Clinical pathologic parameters were measured using a fully automatic hematology analyzer (BC-2800Vet, Mindray, China).

### Cell isolation and flow cytometry

Spleens were ground and filtered by using 100 μm cell strainer (352360, BD Bioscience, USA) in RPMI 1640 containing 10% FBS. Spleen cells were incubated on ice for 30 min with optimal concentrations of anti-mouse CD16/CD32 (clone 2.4G2, BD Bioscience, USA), fluorochrome-conjugated antibodies against anti-mouse Gr-1 (clone RB6-8C5, eBioscience, USA), anti-mouse TER119 (clone TER119, eBioscience, USA), anti-mouse CD11b (clone M1/70, eBioscience, USA), anti-mouse CD11c (clone N418, eBioscience, USA), anti-mouse B220 (clone RA3-6B2, eBioscience, USA), anti-mouse CD4 (clone GK1.5, eBioscience, USA), and anti-mouse CD8 (clone 53–6.7, eBioscience, USA) antibodies. Dead cells were excluded using 7AAD (559925, BD bioscience, USA). Samples were acquired using LSRFortessa (BD Bioscience, USA) and analyzed using FlowJo (BD Bioscience, USA).

### Histological evaluation

The heads of RP mice that had been subjected to RP blood collection were harvested, fixed in 10% neutral-buffered formalin, and embedded in paraffin. Embedded head tissues were sectioned in the coronal plane at a thickness of 3 μm. The sections were then stained with hematoxylin and eosin. Slides were observed using an optical microscope (DM5000B, Leica, Germany).

### Statistical analysis

All experimental results were expressed as the mean ± standard deviation. Data were analyzed using parametric two-way ANOVA or the two-tailed unpaired Student’s t-test. A *P*-value < 0.05 was considered to indicate statistical significance. Graphs were constructed using Prism 8.0 software (GraphPad, USA).

## Data Availability

The datasets supporting the conclusions of this article are available from the corresponding author on reasonable request.

## Abbreviations

RP: Retroorbital plexus
FV: Facial vein
IACUCs: Institutional Animal Care and Use Committees
WBC: White blood cell
